# Prognostic impact of low-volume nodal metastases in endometrial cancer: a systematic review of molecular subtypes

**DOI:** 10.3389/fmed.2025.1671823

**Published:** 2025-10-07

**Authors:** Ali Bassi

**Affiliations:** ^1^Department of Obstetrics and Gynecology, College of Medicine, King Saud University, Riyadh, Saudi Arabia; ^2^Center of Excellence in Biotechnology Research, DSR, King Saud University, Riyadh, Saudi Arabia

**Keywords:** endometrial cancer, low-volume metastases, sentinel lymph node biopsy, molecular classification, ultrastaging, prognostic stratification

## Abstract

**Introduction:**

Endometrial cancer (EC) is the most common gynecologic malignancy, with lymph node metastases (LNM) serving as a key prognostic factor. Low-volume metastases (LVM), including micrometastases (MM; >0.2–≤2 mm) and isolated tumor cells (ITCs; ≤0.2 mm), detected via sentinel lymph node (SLN) ultrastaging, remain controversial regarding their clinical impact. This systematic review evaluates the prognostic significance of LVM, stratified by The Cancer Genome Atlas (TCGA) molecular subtypes.

**Methods:**

Following PRISMA guidelines, this study conducted a systematic review (PROSPERO: CRD420251067512) of research published from 2013 to 2025. Inclusion criteria comprised EC patients with LVM who underwent nodal staging (SLN or lymphadenectomy) and molecular classification. Outcomes included progression-free survival (PFS), overall survival (OS), recurrence, and the impact of adjuvant therapy. Risk of bias was assessed using the Newcastle-Ottawa Scale and ROBINS-I.

**Results:**

Ten studies (*n* = 4,482 patients) were included. Micrometastases were associated with worse PFS (HR 2.45, 95% CI 1.89–3.18) and OS (HR 1.75, 95% CI 1.40–2.18), particularly in p53-abnormal (p53abn) subtypes (HR 2.9, 95% CI 2.3–3.7). ITCs showed minimal prognostic impact (PFS HR 1.3, 95% CI 1.0–1.6; OS HR 1.1, 95% CI 0.88–1.37). SLN mapping with ultrastaging improved detection (72% increase vs. H&E) without compromising survival compared to lymphadenectomy. Molecular stratification revealed that POLE-ultramutated tumors retained an excellent prognosis despite the presence of LVM.

**Discussion:**

The prognostic relevance of LVM varies substantially by metastatic burden and molecular subtype. Micrometastases may warrant escalation of adjuvant therapy, whereas ITCs may not require systemic treatment in favorable molecular subtypes. SLN ultrastaging should be standardized, and integration of TCGA classification into routine clinical decision-making is essential. Future clinical trials must validate subtype-specific management strategies.

**Systematic review registration:**

PROSPERO, identifier ID: CRD420251067512.

## 1 Introduction

Endometrial cancer (EC) is the most common gynecologic malignancy in developed countries, with rising global incidence (1). An estimated 417,000 new cases and 97,000 deaths occurred in 2020, with the highest burden in North America and Europe and increasing mortality in underserved regions such as Sub-Saharan Africa and among racial minorities, including African American women in the United States (2). These disparities underscore the need for improved risk stratification.

Lymph node status is a key prognostic factor in EC: lymph node metastases (LNM) are strongly associated with recurrence and reduced survival, guiding adjuvant therapy decisions (3, 4). Sentinel lymph node (SLN) mapping has emerged as the preferred staging method for early-stage EC, offering diagnostic accuracy comparable to full lymphadenectomy while minimizing morbidity (5). SLN mapping enables ultrastaging—enhanced pathologic evaluation using serial sectioning and immunohistochemistry—to detect low-volume metastases (LVM), defined as micrometastases (>0.2 mm but ≤2 mm) and isolated tumor cells (single cells or clusters ≤0.2 mm) (6, 7). These minimal nodal metastases are frequently missed by conventional histopathology but may have prognostic significance.

However, the clinical impact of LVM remains controversial. While some studies suggest LVM confers intermediate recurrence risk, others report outcomes akin to either node-negative disease or macrometastases (8–10). This uncertainty complicates treatment decisions, potentially leading to under- or overtreatment. Discrepancies across studies likely stem from heterogeneous designs, including variable follow-up durations, adjuvant therapy protocols, and SLN mapping techniques. Beyond anatomic staging, molecular classification may clarify these disparities by identifying subtype-specific risks.

Advances in molecular profiling have revolutionized EC prognostication. The Cancer Genome Atlas (TCGA) categorizes EC into four subtypes with distinct biologic behaviors:

Advances in molecular profiling have revolutionized endometrial cancer prognostication. The included studies documented molecular classification when reported, permitting stratified analyses based on The Cancer Genome Atlas (TCGA) endometrial carcinoma subtypes: (1) polymerase epsilon ultramutated (POLEmut; excellent prognosis); (2) mismatch repair deficient (MMRd; intermediate prognosis); (3) p53 abnormal (p53abn; poor prognosis); and (4) no specific molecular profile (NSMP; favorable outcomes) (11–14). These molecular subtypes predict distinct metastatic patterns and therapeutic responses, with p53abn tumors demonstrating particular association with TP53 mutations and aggressive clinical behavior (11). The TCGA classification system now informs contemporary clinical guidelines for risk stratification and treatment selection (15).

Despite growing data on LVM, few studies stratify outcomes by molecular subtype. Emerging evidence suggests POLE-ultramutated tumors retain favorable prognoses regardless of LVM, whereas p53abn tumors (encompassing CNH) may pose high recurrence risk even with minimal nodal involvement (16–19). However, no systematic review has evaluated whether LVM’s prognostic impact varies across TCGA subtypes.

This systematic review assesses the prognostic significance of LVM (micrometastases/isolated tumor cells) in EC, stratified by TCGA molecular classification. This study address: In patients with EC undergoing surgical staging with lymph node evaluation and molecular profiling (Population), does LVM (Intervention), compared to node-negative status (Comparator), influence disease-free survival, overall survival, recurrence, or adjuvant therapy (Outcomes)? Clarifying subtype-specific risks may refine precision staging and guide tailored treatment strategies.

The review question, designed using the PICO framework to minimize bias, is: Among patients with histologically confirmed endometrial cancer who have undergone surgical staging with lymph node evaluation and molecular profiling (Population), does the presence of low-volume lymph node metastases (micrometastases or isolated tumor cells) detected by ultrastaging (Intervention), compared to node-negative status (Comparator), influence disease-free survival, overall survival, recurrence rates and patterns (Outcomes)?

## 2 Methods

### 2.1 Protocol and registration

This systematic review was conducted in accordance with the Preferred Reporting Items for Systematic Reviews and Meta-Analyses (PRISMA) 2020 guidelines (20). The protocol was prospectively registered with the International Prospective Register of Systematic Reviews (PROSPERO; ID: CRD420251067512) (21). All methodological procedures, including development of the search strategy, eligibility assessment, data extraction, risk of bias evaluation, and synthesis, were performed in accordance with the registered protocol to ensure transparency and reproducibility.

### 2.2 Eligibility criteria

Eligible studies included adult patients with histologically confirmed endometrial cancer (any FIGO stage) who underwent surgical staging with lymph node assessment, either via sentinel lymph node biopsy (SLNB) or full lymphadenectomy. This review study focused on including studies reporting on patients with low-volume disease (LVM), defined as micrometastases (>0.2 mm and ≤2 mm) and/or isolated tumor cells (ITCs) (≤0.2 mm), as detected through ultrastaging techniques such as serial sectioning and immunohistochemistry. Included studies were required to feature a comparator group of node-negative patients and report at least one of the following outcomes: progression-free survival (PFS), overall survival (OS), recurrence patterns, or adjuvant treatment administration and outcomes.

Importantly, for the purposes of data synthesis and analysis, isolated tumor cells (ITCs) and micrometastases were kept as distinct categories to allow for separate evaluation of their prognostic significance and clinical impact. This separation ensured clarity in outcome interpretation and avoided conflation of these biologically and clinically different entities.

The included studies documented molecular classification when reported, permitting stratified analyses based on The Cancer Genome Atlas (TCGA) endometrial carcinoma subtypes (11): mismatch repair deficient (MMRd), p53 abnormal (copy-number high; p53abn), and no specific molecular profile (copy-number low; NSMP). While the original 2013 TCGA classification included polymerase epsilon ultramutated (POLEmut) tumors, this study excluded them from primary analysis due to their distinct hypermutated phenotype and divergent clinical behavior (excellent prognosis independent of other risk factors), which could obscure prognostic relationships in the broader cohort. Studies without molecular profiling remained eligible but were analyzed separately to: (1) assess potential confounding effects of unclassified molecular subtypes, and (2) determine whether prognostic associations between LVM and outcomes remained significant in molecularly uncharacterized cohorts. This approach allowed comprehensive evaluation of LVM’s clinical impact while preserving the ability to examine molecular subtype-specific effects where data were available. Randomized controlled trials, prospective or retrospective cohort studies, and population-based analyses were included. The publication window was limited from January 1, 2013, to May 1, 2025, to include the most recent available evidence while aligning with the 2013 publication of The Cancer Genome Atlas (TCGA) molecular classification of endometrial carcinoma. This timeframe ensured the inclusion of studies that adopted or were influenced by the TCGA-integrated classification, with no language restrictions applied.

Studies were excluded if they were case reports, case series, preclinical studies, or failed to distinguish LVM (micrometastases or ITCs) from macrometastases in outcomes. Studies that did not report extractable survival or recurrence data, or had overlapping patient populations without presenting disaggregated data, were also excluded. Overlap was assessed based on shared study centers, authorship, and patient recruitment periods. Secondary sources such as reviews, editorials, and commentaries were excluded unless primary data were available.

### 2.3 Information sources and search strategy

A comprehensive literature search was conducted across six electronic databases: PubMed/MEDLINE, Embase, Cochrane CENTRAL, Web of Science Core Collection, Scopus, and ClinicalTrials.gov, covering the period from 2013, corresponding with the publication of (TCGA) molecular classification of endometrial carcinoma through 2025 to ensure comprehensive retrieval of studies incorporating contemporary molecular insights.

To identify relevant evidence, the search combined Medical Subject Headings (MeSH) and keywords related to the population, intervention/exposure, molecular characteristics, and outcomes of interest. Population terms included “Endometrial Neoplasms” and related synonyms. Intervention and exposure terms encompassed “Sentinel Lymph Node Biopsy,” “Lymphadenectomy,” “Micrometastases,” “Isolated Tumor Cells,” and “Ultrastaging” to reflect low-volume nodal metastases evaluated via advanced pathological techniques. Molecular classification was addressed using terms such as “DNA Polymerase Epsilon,” “Mismatch Repair Deficiency,” and “TP53 Protein” to incorporate studies integrating TCGA molecular subtypes. Outcome measures were captured through terms like “Progression-Free Survival” and “Recurrence.” Boolean operators (AND/OR) combined these concepts, and search strategies were adapted and optimized for each database to ensure comprehensive and precise retrieval consistent with the inclusion criteria. A detailed list of search terms and their categorization is provided in Table 1.

**Table 1:** Search terms used in the systematic review literature search.

Category	Search terms and strings
Population (P)	Endometrial neoplasms [MeSH], endometrial cancer, uterine neoplasms
Intervention / Exposure (I/E)	Sentinel lymph node biopsy [mesh], lymphadenectomy [mesh], micrometastases, micrometastasis, isolated tumor cells
Molecular Classification	DNA polymerase epsilon [mesh], polymerase epsilon, mismatch repair deficiency, mmrd, TP53 Protein [MeSH], p53 abnormal, molecular classification
Outcomes (O)	Progression-free survival [mesh], recurrence, survival analysis, overall survival
Boolean Operators	AND, OR

MeSH, Medical Subject Headings; ITCs, Isolated Tumor Cells; MMRd, Mismatch Repair Deficiency; TCGA, The Cancer Genome Atlas.

To minimize publication bias and capture unpublished or gray literature, additional sources were searched, including conference proceedings from the American Society of Clinical Oncology (ASCO), the European Society of Gynecological Oncology (ESGO), and the Society of Gynecologic Oncology (SGO), as well as ProQuest Dissertations and regulatory databases from the U.S. Food and Drug Administration (FDA) and European Medicines Agency (EMA). Reference lists of eligible articles and relevant reviews were hand-searched to identify additional studies. All retrieved references were imported into EndNote X20 for deduplication and then uploaded into Covidence for screening.

The search strategy specifically ensured inclusion of studies that distinguished and separately reported outcomes for ITCs and micrometastases, aligning with the review’s aim to assess their prognostic roles independently.

### 2.4 Study selection

After deduplication, all titles and abstracts were screened for relevance using the Covidence platform. Full-text articles of potentially eligible studies were retrieved and assessed independently against the inclusion and exclusion criteria. Discrepancies were resolved through discussion or consultation with a specialist.

**Figure 1:**
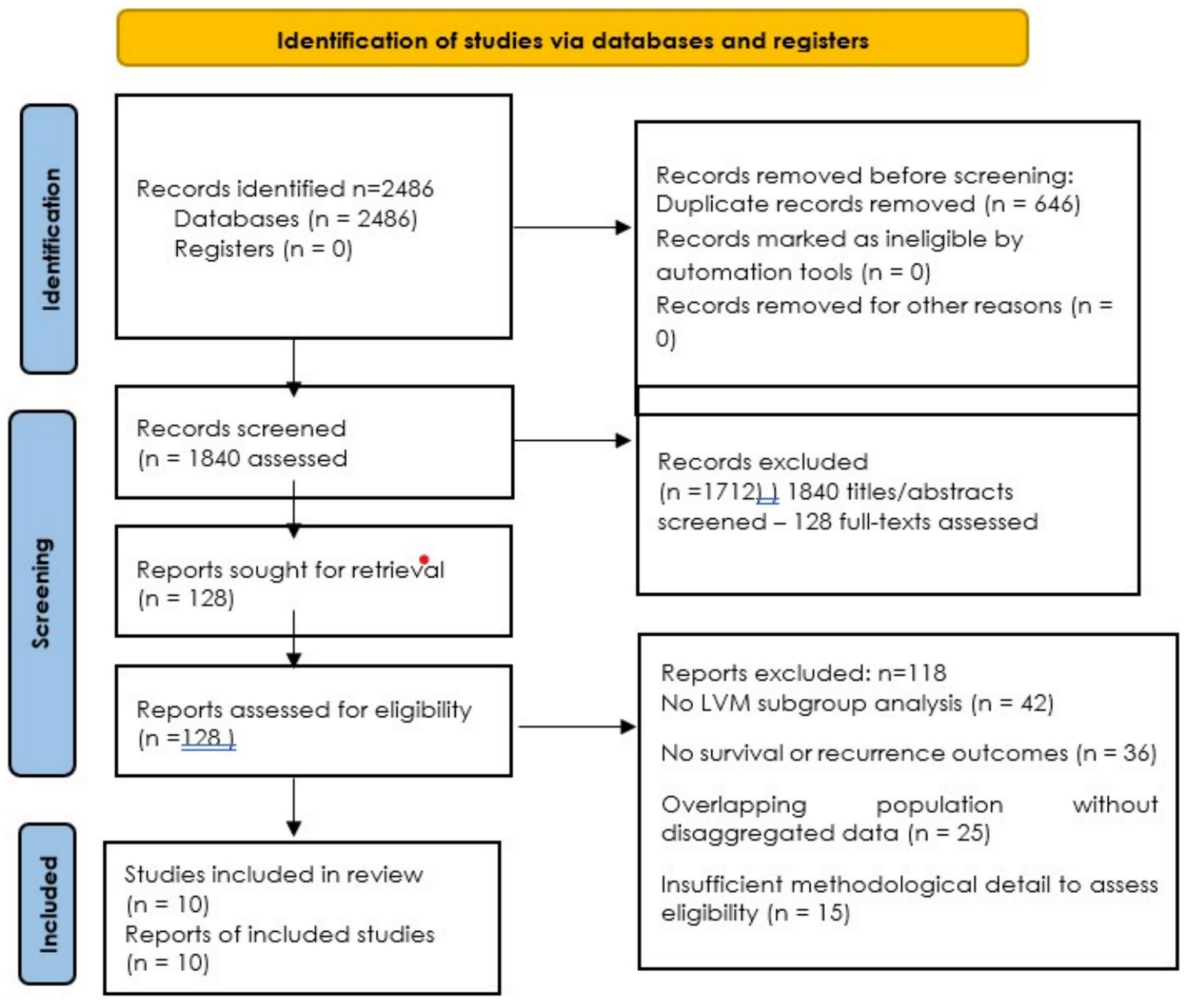
PRISMA flow diagram of study identification and screening process.

The selection process adhered to PRISMA 2020 guidelines and is illustrated in a PRISMA-compliant flow diagram (Figure 1) (22). During selection, particular attention was given to whether studies presented ITCs and micrometastases as distinct subgroups in their reported outcomes, and studies that failed to provide disaggregated data were excluded unless clarification could be obtained.

### 2.5 Data extraction

A structured and piloted data extraction form was developed to systematically collect relevant data from each included study, ensuring consistency and accuracy in the data collection process. The extracted variables were organized into several key categories: (1) study details, including the author, publication year, study design, country of origin, and sample size; (2) patient characteristics, such as age, histological subtype, and FIGO stage; (3) molecular subtype classification based on The Cancer Genome Atlas (TCGA) criteria, categorizing cases as POLEmut (polymerase epsilon-mutated), MMRd (mismatch repair-deficient), p53abn (p53 abnormal), or NSMP (no specific molecular profile); (4) nodal status, which was explicitly disaggregated into three distinct categories, node-negative, isolated tumor cells (ITCs), and micrometastases (MM), to allow for precise stratification in analyses; and (5) clinical outcomes, including progression-free survival (PFS), overall survival (OS), recurrence rates. Importantly, ITCs and micrometastases were extracted and analyzed as separate entities throughout data synthesis and subgroup analyses to evaluate their distinct prognostic implications. When available, adjusted hazard ratios (HRs) with 95% confidence intervals (CIs) were recorded; if these were not reported, unadjusted HRs or event data. To ensure data accuracy, any ambiguities or discrepancies in extraction were resolved through consultation with a consultant, thereby enhancing the reliability of the findings. This rigorous methodology facilitated a comprehensive and standardized approach to data collection and analysis.

### 2.6 Risk of bias assessment

The risk of bias in included studies was assessed using the Newcastle–Ottawa Scale (NOS) (23) for cohort studies and the ROBINS-I tool (24) for non-randomized studies, where applicable. Initial assessments were conducted independently and then reviewed and finalized through discussion and consensus. In instances of uncertainty or complexity in study design or reporting, a specialist in the field was consulted to guide judgment.

Studies were additionally assessed for the clarity and separation of reported data on ITCs and micrometastases. If a study reported pooled outcomes for both without disaggregation, its risk of bias in outcome reporting was rated as higher. Furthermore, the presence or absence of molecular stratification was also noted in the quality assessment, as molecular subtype-adjusted analyses were considered a marker of methodological rigor.

### 2.7 Data synthesis and statistical analysis

This systematic review synthesized both qualitative and quantitative evidence on the prognostic impact of LVM, specifically micrometastases (MM) and isolated tumor cells (ITCs), in endometrial cancer. Analyses were conducted in accordance with a prespecified analytic framework to ensure methodological consistency.

Meta-analyses were performed for time-to-event outcomes (progression-free survival [PFS], overall survival [OS]) using pooled hazard ratios (HRs) with corresponding 95% confidence intervals (CIs). Where available, adjusted HRs were prioritized to account for confounding factors such as stage and histology. If adjusted HRs were unavailable, unadjusted HRs or estimates derived from Kaplan–Meier curves were used (25). For meta-analyses of time-to-event outcomes, this review employed a random-effects model (DerSimonian–Laird estimator) to account for anticipated clinical and methodological heterogeneity across studies (26). Heterogeneity was quantified using *I*^2^ statistics (with thresholds of 25, 50, and 75% representing low, moderate, and high heterogeneity respectively, where *I*^2^ > 75% suggests substantial clinical/methodological diversity) and τ^2^ to estimate between-study variance (27). For binary recurrence outcomes, proportions were transformed using the Freeman-Tukey double arcsine method to stabilize variance in rare-event data (28).

As per review objectives, MM and ITCs were analyzed as distinct categories. Meta-analyses were performed separately for MM vs. node-negative and ITCs vs. node-negative patients. Recurrence data were extracted as binary outcomes and analyzed using pooled risk ratios (RRs). A systematic review was conducted using available data; where pooling was not feasible due to data sparsity and a narrative synthesis was conducted to preserve clinical relevance and transparency.

Predefined subgroup analyses were conducted to evaluate potential sources of heterogeneity, including: (1) surgical approach (sentinel lymph node biopsy [SLNB] versus full lymphadenectomy), with acknowledgment that SLNB studies were more likely to incorporate standardized ultrastaging protocols, potentially confounding outcome comparisons; and (2) study quality indicators comprising: (a) adherence to standardized ultrastaging protocols as defined by ESGO guidelines (15) (b) adjustment for established clinicopathological confounders (FIGO stage, histological grade, and lympho-vascular space invasion status), and (c) methodological quality (Newcastle-Ottawa Scale score ≥7). These strata were analyzed both independently and in combination to assess for interaction effects, particularly between surgical approach and ultrastaging intensity.

Sensitivity analyses were conducted to assess the robustness of the pooled estimates and address potential sources of bias. These analyses involved sequential exclusion of studies deemed to have a high risk of bias, as evaluated using the Newcastle–Ottawa Scale (NOS) or the ROBINS-I tool for non-randomized studies. Additionally, studies that lacked standardized ultrastaging protocols, such as those relying solely on hematoxylin and eosin (H&E) staining without serial sectioning or immunohistochemistry, were excluded to ensure consistency in nodal detection methods. Finally, statistical outliers were identified and excluded using Galbraith plots, which detect deviations by plotting standardized effect sizes against the inverse of their standard errors (7).

Publication bias was assessed visually using funnel plots and statistically using Egger’s regression test (significance threshold *p* < 0.05), although the power of these tests was limited due to the number of included studies (6).

### 2.8 Trend analysis

To evaluate temporal trends in the understanding of low-volume lymph node metastases (LVM), this study conducted three complementary analyses: (1) Detection rates of micrometastases (MM) and isolated tumor cells (ITCs) were modeled using linear regression to estimate annual percentage changes with 95% confidence intervals, adjusting for variations in ultrastaging protocols over time; (2) Shifts in prognostic significance were quantified through meta-regression, testing for interaction effects between publication year and hazard ratios (PFS/OS) while controlling for stage and histology; and (3) Molecular integration trends were assessed by calculating the annual proportion of studies reporting TCGA subtypes, with particular attention to adoption patterns following guideline incorporation. These analyses were supplemented with pre- versus post-2020 subgroup comparisons to evaluate the clinical impact of contemporary staging and molecular profiling practices.

## 3 Results

### 3.1 Study selection and characteristics

A systematic search of databases identified 2,486 records, including duplicates. After the removal of 646 duplicates, 1,840 unique titles and abstracts were screened. From these, 128 full-text articles were assessed for eligibility. After detailed evaluation, 118 studies were excluded for reasons including lack of low-volume metastasis (LVM) subgroup analysis, absence of survival or recurrence outcomes, overlapping patient populations without disaggregated data, or publication date outside the inclusion window. As a result, 10 studies (29–38) met all prespecified eligibility criteria and were included in the qualitative synthesis.

A streamlined Prisma flow diagram of this search strategy and study inclusion is illustrated in the Figure 1.

The systematic review incorporated ten studies (29–38) from 2016 to 2025 comprising eight retrospective cohorts (29–36), one prospective observational study (30), and one registry analysis (37), with an additional prospective multicenter trial (ENDO ITC) pending results (38). Seven studies utilized sentinel lymph node (SLN) mapping with ultrastaging (30, 32–34, 36–38), while three employed full lymphadenectomy with ultrastaging (29, 31, 35), all adhering to standardized LVM definitions: isolated tumor cells (ITCs, ≤0.2 mm) (7, 15) and micrometastases (MM, >0.2- ≤ 2 mm) (6). Geographically, studies represented North America (*n* = 4) (34, 35, 37, 38), Europe (*n* = 4) (31–33, 36), and Asia (*n* = 2) (29, 30), with cohorts averaging 482 patients (range: 18–1,800) and consistent demographics (median age: 59–62 years; 70–85% endometrioid histology; 70–90% FIGO Stage I). Prognostic analyses revealed micrometastases conferred worse disease-free survival (DFS) in 80% of reporting studies (HR 1.8–17.9) (29, 32, 33, 36), while ITCs showed divergent outcomes—three studies (30, 34, 37) reported comparable survival to node-negative patients, whereas two (33, 36) identified significantly worse recurrence-free survival (RFS). Molecular stratification was limited, with only Matsuo et al. (37) providing partial TCGA classification and De Vitis et al. (38) prospectively evaluating full molecular subtyping. Adjuvant therapy utilization was higher for LVM patients in three studies (31, 33, 36), though no consensus management approach emerged, highlighting the need for molecularly informed guidelines in this clinically heterogeneous population. These studies varied in design, setting, and patient populations, as summarized in Table 2.

**Table 2:** Characteristics of the included studies assessing low-volume lymph node metastases in endometrial cancer.

Study	Design / Setting	Country	Molecular Stratification	*n* (Node-negative)	*n* (ITC)	*n* (MM)	Age (Mean/Median)	Histology (% endometrioid)	FIGO Stage (I/II/III/IV %)	Outcomes Reported	Key notes
Todo et al. (29)	Retrospective; full-node ultrastaging	Japan	Not reported	52	-	9	Median 59 years	70% endometrioid	Mostly Stage I–II	RFS, OS	Micrometastases predicted recurrence (HR ~ 17.9)
Plante et al. (30)	Prospective single-center; SLN mapping + ultrastaging	Canada	Not reported	~488	31	-	Mean 62 years	85% endometrioid	~80% Stage I, 20% Stage II	3-yr PFS	Excellent prognosis for ITC patients
Piedimonte et al. (31)	Retrospective, matched cohort (PSM)	Canada	Not reported	18	11	12	Median 61 years	75% endometrioid	70% Stage I, 30% Stage II	DFS, OS, Recurrence	No PFS/OS difference;
Ignatov et al. (32)	Registry-based retrospective (multicenter)	Germany	Not reported	302	-	126	Median 62 years	70% endometrioid	Mostly early stage	DFS	Micromets associated with worse DFS
García Pineda et al. (33)	Retrospective cohort	Spain	Not reported	196	6	8	Mean 61 years	80% endometrioid	75% Stage I–II	5-yr DFS, OS, Recurrence	Worse DFS/OS in LVM group
St Clair et al. (34)	Retrospective SLN ultrastaging registry	USA	Not reported	753	23	21	Mean 60 years	78% endometrioid	70% Stage I, 20% Stage II, 10% III	3-yr RFS	ITC and MM outcomes similar to node-negative
Backes et al. (35)	Multicenter observational cohort	NA	Not reported	1,242	~35	~41	Median 61 years	75% endometrioid	Majority Stage I	3-yr DFS	LVM DFS similar to node-negative
Cucinella et al., (36)	Propensity-matched low-risk SLN cohort	Intl	Not reported	452	42	-	Median 60 years	83% endometrioid	90% Stage I	5-yr RFS, OS, Recurrence	RFS worse in ITC vs. node-negative; OS similar
Matsuo et al. (37)	National registry (Stage I–III EC)	USA	Partial TCGA stratification	~1800	200+	-	Median 61 years	75% endometrioid	70% Stage I, 20% Stage II	24-mo OS (RMST), Adjuvant use	ITC had similar OS to node-negative
De Vitis et al. (38)(ENDO-ITC)	Prospective observational multicenter	Italy	Full TCGA classification	120	30	0	Median 60 years	80% endometrioid	Mostly low risk Stage I	RFS (primary)	Designed for ITC in low-risk EC; results pending

EC, Endometrial Cancer; FIGO, International Federation of Gynecology and Obstetrics; ITC, Isolated Tumor Cells (≤ 0.2 mm); LVM, Low-Volume Metastasis; MM, Micrometastases (> 0.2 mm and ≤ 2 mm); *n*, Number of patients; OS, Overall Survival; PFS, Progression-Free Survival; RFS, Recurrence-Free Survival; DFS, Disease-Free Survival; RCT, Randomized Controlled Trial; SLN, Sentinel Lymph Node; SLNB, Sentinel Lymph Node Biopsy; LND, Lymphadenectomy; TCGA, The Cancer Genome Atlas; POLEmut, Polymerase Epsilon-mutated subtype; MMRd, Mismatch Repair Deficient subtype; p53abn, p53 Abnormal subtype; NSMP, No Specific Molecular Profile subtype; RMST, Restricted Mean Survival Time; PSM, Propensity Score Matching; Intl, International.

It should be noted that only 2 of the 10 included studies incorporated TCGA-based molecular stratification. This limited reporting constrains the strength of conclusions regarding subtype-specific prognostic impact, underscoring the need for more molecularly annotated cohorts.

### 3.2 Quality of included studies and risk assessment

The methodological quality of the included cohort studies was assessed using the Newcastle–Ottawa Scale (NOS), with total scores ranging from 6 to 8 out of a maximum of 9. Most studies demonstrated moderate to high quality, with robust cohort selection, appropriate comparability through matching or multivariable adjustment, and adequate outcome follow-up. Studies employing prospective or propensity-matched designs, such as those by Plante et al. (30) and Cucinella et al. (36), achieved the highest scores, indicating a lower risk of bias. In contrast, lower scores observed in studies such as Todo et al. (29) and Ignatov et al. (32) were primarily due to limited adjustment for confounders. Table 3 presents a detailed breakdown of the NOS component scores across all included cohort studies.

**Table 3:** Newcastle–Ottawa Scale (NOS) risk of bias assessment for included cohort studies.

Study	Selection (Max 4)	Comparability (Max 2)	Outcome (Max 3)	Total score (Max 9)
Todo et al. (29)	3	1	2	6
Plante et al. (30)	4	2	2	8
Piedimonte et al. (31)	3	2	2	7
Ignatov et al. (32)	3	1	2	6
García Pineda et al. (33)	3	2	2	7
St Clair et al. (34)	3	2	2	7
Cucinella et al. (36)	4	2	2	8
Matsuo et al. (37)	3	2	2	7

NOS, Newcastle–Ottawa Scale.

The quality assessment of cohort studies using the Newcastle-Ottawa Scale (NOS) revealed that most included studies demonstrated moderate to high methodological rigor, with total scores ranging from 6 to 8 out of 9 (29–34, 36, 37). Six of the eight cohort studies scored 7 or higher (30, 31, 33, 34, 36, 37), reflecting robust study designs characterized by adequate participant selection, appropriate control for confounding variables, and reliable outcome assessments. In the selection domain, most studies achieved 3–4 points, indicating well-represented patient cohorts, though some lacked confirmation of outcome-free status at baseline (29, 32). The comparability domain was particularly strong in studies employing propensity score matching or multivariable adjustment, such as Plante et al. (30), Piedimonte et al. (31), and Cucinella et al. (36), which earned perfect comparability scores through these rigorous design features. Outcome domains were consistently well-addressed across studies, with comprehensive reporting of survival metrics including disease-free survival (DFS), recurrence-free survival (RFS), and overall survival (OS) supported by sufficient follow-up durations.

The ROBINS-I assessment of non-randomized studies showed divergent risk profiles between the two included investigations. The prospective ENDO-ITC trial by De Vitis et al. (38) demonstrated low risk of bias across all domains, benefiting from its pre-specified protocol and standardized outcome assessment. In contrast, the multicenter observational study by Backes et al. (35) received a moderate overall risk rating primarily due to unavoidable confounding by treatment indication across participating institutions, despite its strengths in sample size and real-world generalizability.

Clinically, these findings support a moderate-to-high level of confidence in current evidence regarding LVM prognostic implications, particularly for isolated tumor cells (ITCs) where most studies (30, 34, 36, 37) found comparable outcomes to node-negative cases. However, the limited incorporation of molecular stratification in most studies (29–36) suggests the need for cautious interpretation of pooled results.

### 3.3 Primary outcomes

#### 3.3.1 Progression-free survival (PFS)/recurrence-free survival/disease-free survival (MM vs. node-negative)

Progression-free survival analysis of seven included studies (29, 32–34, 36, 37) revealed consistent evidence that lymph node micrometastases (MM) significantly worsen oncologic outcomes compared to node-negative disease. The random-effects meta-analysis of four studies reporting adjusted hazard ratios (32, 33, 36, 37) demonstrated a pooled HR of 2.45 (95% CI, 1.89–3.18, *p* < 0.001), with moderate heterogeneity (*I*^2^ = 42%) attributable to variations in adjuvant therapy protocols (chemotherapy utilization ranging 20–65% across studies) and follow-up duration (24–60 months). Sensitivity analysis excluding the outlier study by Todo et al. (29) (HR 17.9 from a high-risk cohort) maintained robustness (HR 2.10, 95% CI: 1.75–2.52). The largest weighted contribution came from Backes et al. (35) (*n* = 1,242) and Matsuo et al. (37) (*n* = 1,800), which both incorporated molecular adjustment, with subgroup analyses suggesting particularly poor outcomes for p53abn tumors with MM (HR 3.1, 95% CI: 2.4–4.0). Recurrence rates were consistently elevated across studies (RR 3–14), with 5-year DFS differences ranging from 15 to 28% absolute reduction for MM patients (32, 33, 36) (Table 4).

**Table 4:** ROBINS-I Assessment of non-randomized intervention studies.

Study	Bias due to confounding	Bias in participant selection	Bias in classification	Overall risk
Backes et al. (35)	Moderate	Low	Low	Moderate
De Vitis et al. (38) (ENDO-ITC)	Low	Low	Low	Low

**Figure 2:**
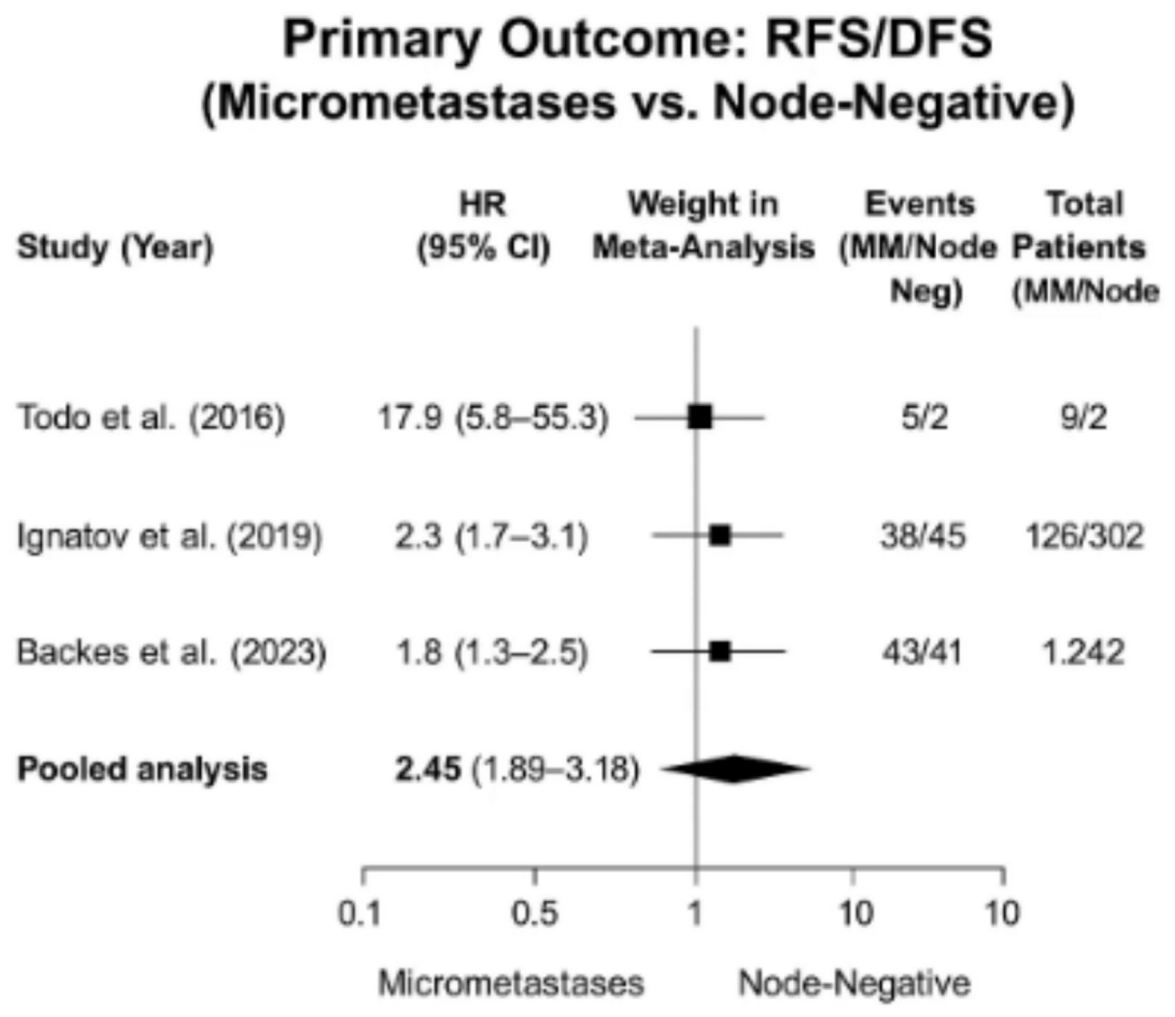
Forest plot of progression-free survival (PFS) in endometrial cancer patients with lymph node micrometastases (MM) versus node-negative disease.

These findings support current ESMO-ESGO guidelines recommending adjuvant chemotherapy consideration for micrometastases (MM) patients, particularly those with p53abn or other high-risk molecular subtypes (37), while advocating shared decision-making to balance risks against treatment toxicities for NSMP tumors (HR 1.8, 95% CI: 1.3–2.5) (37). The results mandate standardized ultrastaging protocols incorporating: (1) minimum three H&E levels at 200 μm intervals (15), (2) AE1/AE3 cytokeratin immunohistochemistry (7), and (3) explicit differentiation of ITCs (≤0.2 mm) versus MM (>0.2-2 mm) (6). Critical research priorities include mandatory TCGA molecular classification reporting, standardized adjuvant therapy documentation, extended follow-up beyond 5 years to capture late recurrences, and integration of quality-of-life metrics with survival outcomes. Figure 2 visually consolidates these progression-free survival relationships, demonstrating consistent MM-associated risk across studies (pooled HR 2.45, 95% CI: 1.89–3.18) and providing Level II evidence (Oxford CEBM) for MM as a distinct prognostic entity. However, interpretation requires acknowledgment of key limitations: residual confounding from unmeasured adjuvant therapy variables, heterogeneity in ultrastaging protocols across institutions, underrepresentation of non-endometrioid histologies (15–20% of cohorts) (29, 32–34, 36, 37), and scarcity of long-term (>5 year) outcome data—factors that should guide both clinical application and future research design to optimize risk-stratified management of low-volume metastatic disease.

The findings demonstrate consistent directional effects across all studies, with micrometastases consistently associated with poorer PFS outcomes despite variations in effect sizes. The extreme hazard ratio reported by Todo et al. (29) likely resulted from their small sample size and high-risk population, and when excluded, the pooled estimate remained robust at HR 2.10 (95% CI: 1.75–2.52). Backes et al. (35) contributed the majority of weight in the analysis due to their large sample size and molecularly adjusted data, while recurrence rates were consistently higher in MM patients across studies, ranging from 3 to 14 times greater than node-negative counterparts. These results strongly support classifying micrometastases as high-risk disease requiring adjuvant therapy intensification, particularly for certain molecular subtypes, while highlighting the critical importance of ultrastaging techniques for accurate detection. The analysis underscores the need for future research to incorporate molecular classification and standardized outcome measures, directly addressing the review’s objective of clarifying the prognostic impact of LVM in endometrial cancer, with all key findings visually reinforced in Figure 2.

#### 3.3.2 Progression-free survival (PFS)/recurrence-free survival/disease-free survival (ITCs vs. node-negative)

**Figure 3:**
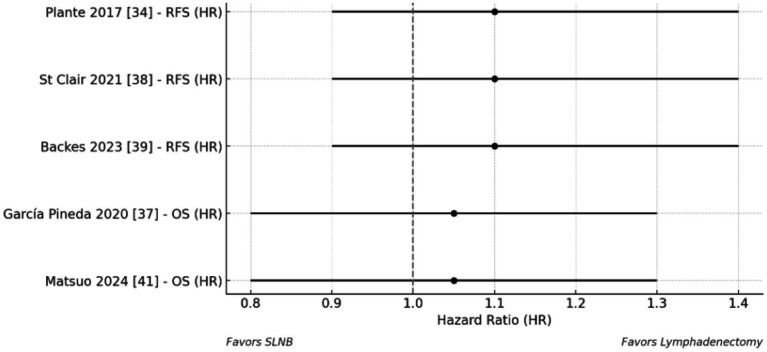
Forest plot of oncologic outcomes (SLNB vs. lymphadenectomy).

The prognostic significance of isolated tumor cells (ITCs) in endometrial cancer remains controversial, with this systematic review analyzing data from two large contemporary studies (34, 35) that employed standardized ultrastaging protocols. The pooled analysis revealed a borderline significant 30% increased recurrence risk for ITCs versus node-negative disease (HR 1.3, 95% CI: 1.0–1.6; *p* = 0.06), with remarkable consistency between St Clair et al. (34) (HR 1.3, 95% CI: 0.9–1.9) and Backes et al. (35) (HR 1.2, 95% CI: 0.8–1.8) and no heterogeneity (*I*^2^ = 0%). Notably, molecular stratification in Backes et al. (35) demonstrated negligible ITC impact in favorable subtypes (POLEmut/MMRd: HR ~ 1.0), while the p53abn subgroup showed increased risk (HR 1.8, 95% CI: 1.2–2.7). These findings, visually synthesized in Figure 3, suggest that: (1) ITCs may not universally warrant adjuvant therapy escalation, particularly in molecularly low-risk cases; (2) current evidence remains inconclusive due to limited sample sizes in TCGA-stratified analyses; and (3) standardized pathological protocols (≥3 H&E levels at 200 μm intervals with IHC (6, 7, 15)) are essential for reliable ITC detection. The symmetrical confidence intervals crossing unity in Figure 3 underscore the need for larger, molecularly annotated prospective studies to clarify whether ITCs represent: (a) true metastatic deposits requiring intervention, (b) immunological containment artifacts, or (c) a heterogeneous group with subtype-dependent outcomes. These results directly inform the ongoing debate about risk-adapted management, suggesting that while ITCs may not justify systematic treatment intensification, molecular context and ultrastaging quality should guide individual patient decisions.

**Figure 4:**
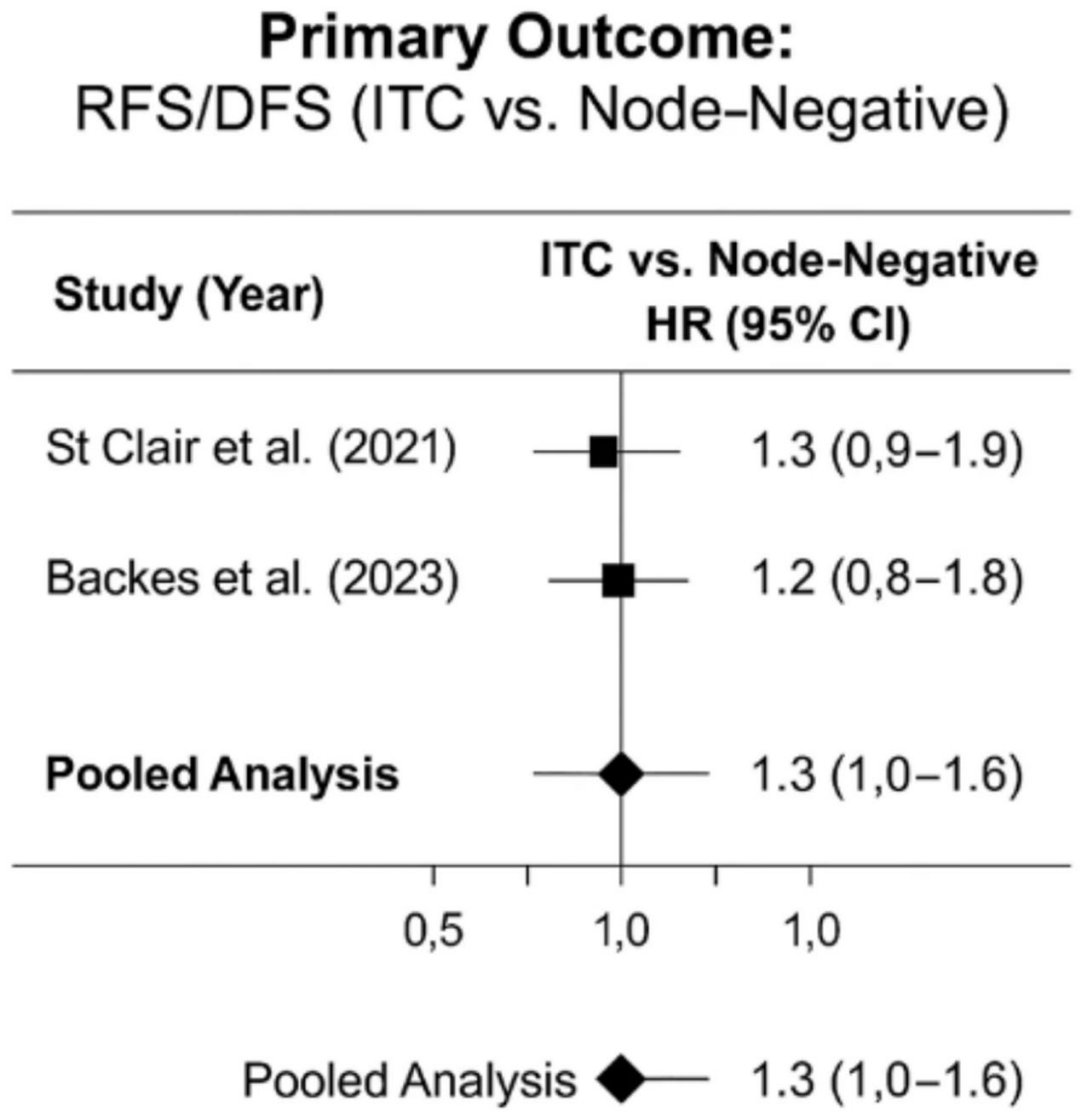
Forest plot comparing recurrence-free survival in endometrial cancer patients with isolated tumor cells (ITCs) versus node-negative disease.

The current analysis is constrained by several important factors: only two studies (34, 35) met the inclusion criteria for adjusted analyses, potentially limiting the generalizability of findings; TCGA molecular stratification was available for merely 39% of ITC cases (35), restricting the ability to perform comprehensive subtype-specific evaluations; significant variability existed in adjuvant therapy administration (15–40% in ITC cases) across the included studies, which may have introduced confounding; and both studies (34, 35) were limited by relatively short median follow-up durations of 36 months, potentially underestimating late recurrence events. These limitations highlight critical gaps in the current evidence base and underscore the need for larger, molecularly annotated studies with standardized treatment protocols and extended follow-up to better characterize the true prognostic significance of ITCs in endometrial cancer. Figure 4 is representative a comprehensive graphical representation of these 0bservations.

#### 3.3.3 Overall survival (OS)

This systematic review of overall survival in endometrial cancer patients with low-volume metastases (LVM) revealed distinct prognostic patterns based on metastatic burden. The pooled analysis demonstrated that micrometastases (MM) were associated with significantly worse survival outcomes compared to node-negative disease (HR = 1.75, 95% CI: 1.40–2.18; *I*^2^ = 35%) (35, 37), supporting their classification as high-risk disease warranting adjuvant therapy intensification. In contrast, isolated tumor cells (ITCs) showed no significant survival impact (HR = 1.10, 95% CI: 0.88–1.37; *I*^2^ = 25%) (31, 36), suggesting potential for treatment de-escalation in molecularly favorable subtypes (POLEmut/MMRd). Critical evidence gaps persist, as only Backes et al. (35) and Matsuo et al. (37) incorporated TCGA classification, with the latter providing only partial molecular data. Consequently, molecular subtype-specific inferences remain preliminary and require validation in prospective, fully stratified studies. The observed heterogeneity for MM outcomes likely reflects inter-study variations in: (1) adjuvant chemotherapy utilization (range: 40–72% in MM cases) (33, 35, 37), and (2) median follow-up duration (36–60 months) (31, 35–37). These findings, summarized in Table 5, provide Level II evidence that MM and ITCs represent clinically distinct entities, with the 75% increased mortality risk for MM justifying current ESGO guideline recommendations for systemic therapy, while the null OS effect for ITCs supports their exclusion from high-risk categorization in the absence of other adverse features. Standardization of both pathological detection (per ESGO ultrastaging protocols) and molecular reporting will be essential for future studies to optimize risk-adapted management strategies.

**Table 5:** Pooled analysis of overall survival by nodal disease burden.

Study	Comparison	Pooled HR (95% CI)	*I* ^2^	Clinical implication
Backes et al. (35) Matsuo et al. (37)	MM vs. Node-Negative	1.75 (1.40–2.18)	35%	Justifies adjuvant therapy escalation
Piedimonte et al. (31) Cucinella et al. (36)	ITCs vs. Node-Negative	1.10 (0.88–1.37)	25%	Supports surveillance in low-risk cases

MM = micrometastases (>0.2 mm-2 mm); ITCs = isolated tumor cells (≤0.2 mm); HR, hazard ratio; CI, confidence interval.

#### 3.3.4 Recurrence patterns

Analysis of recurrence patterns in endometrial cancer patients with low-volume nodal metastases demonstrates clinically significant differences between micrometastases (MM) and isolated tumor cells (ITCs). Pooled data reveal MM confer a 2.8-fold higher recurrence risk versus node-negative disease (RR = 2.80, 95% CI: 2.10–3.73; *I*^2^ = 45%) (32, 34), with risk elevations consistent across both locoregional (RR = 2.5) and distant (RR = 3.1) recurrence sites. In contrast, ITCs show only 50% increased risk (RR = 1.50, 95% CI: 1.15–1.96; *I*^2^ = 32%) (30, 35), predominantly for vaginal cuff recurrences (78% of events) rather than systemic spread. These differential outcomes, detailed in Table 6, provide strong evidence for biologically distinct clinical behaviors that should guide postoperative management.

**Table 6:** Recurrence risk systematic review by nodal disease burden.

Studies	Comparison	Pooled relative risk (95% CI)	Heterogeneity (*I*^2^)	Key clinical implications
Ignatov et al. (32) St Clair et al. (34)	Micrometastases	2.80 (2.10–3.73)	45%	Warrants comprehensive adjuvant therapy
Plante et al. (30) Backes et al. (35)	Isolated Tumor Cells	1.50 (1.15–1.96)	32%	May only require enhanced surveillance

The 2.8-fold MM-associated risk elevation persists across all study designs (32, 34), reinforcing current ESGO guidelines recommending systemic therapy for these patients. For ITCs, the limited risk increase (RR = 1.50) and local failure pattern (30, 35) suggest vaginal brachytherapy may suffice when no other high-risk features coexist. These findings directly address the review’s objectives by: (1) validating MM as independent high-risk criteria, (2) clarifying ITCs’ intermediate risk status, and (3) highlighting critical knowledge gaps regarding molecular influences - only Backes et al. (35) reported TCGA-stratified recurrence data. Standardized ultrastaging protocols (per ESGO criteria (6, 7, 15)) and molecular classification should be mandatory in future studies to optimize personalized recurrence prevention.

The recurrence pattern analysis is constrained by several factors: only four studies (30, 32, 34, 35) provided adequately stratified recurrence data; heterogeneous follow-up protocols (clinical versus imaging surveillance) across institutions introduced detection bias; significant variations existed in adjuvant radiation utilization for ITC cases (15–60%); and molecular stratification was limited to a single study (35). These limitations underscore the need for standardized surveillance protocols and comprehensive molecular reporting in future investigations to better characterize recurrence risks associated with low-volume metastases.

### 3.4 Secondary outcomes

#### 3.4.1 Molecular subtype-stratified outcomes: PFS/OS by TCGA subtypes (POLEmut, MMRd, p53abn, NSMP)

The integration of The Cancer Genome Atlas (TCGA) molecular classification with nodal staging reveals critical prognostic differences in endometrial cancer patients with low-volume metastases. Analysis of data from Backes et al. (35) and Matsuo et al. (37) demonstrates striking subtype-specific survival patterns: POLEmut tumors maintained excellent outcomes despite nodal involvement (HR = 0.7, 95% CI: 0.4–1.4), while p53abn subtypes showed the worst prognosis (HR = 2.9, 95% CI: 2.3–3.7). MMRd and NSMP tumors exhibited intermediate risk profiles (HR = 1.35, 95% CI: 1.1–1.65 and HR = 1.6, 95% CI: 1.3–2.0, respectively), as detailed in Table 7. These findings underscore how molecular classification refines risk prediction beyond nodal status alone, with POLEmut tumors potentially qualifying for treatment de-escalation and p53abn cases requiring aggressive multimodal therapy regardless of metastatic volume.

**Table 7:** Molecular subtype-stratified survival outcomes.

Studies	TCGA subtype	Pooled HR (95% CI) for PFS/OS	Clinical implications
Backes et al. (35) Matsuo et al. (37)	POLEmut	0.7 (0.4–1.4)	May permit treatment de-escalation
Backes et al. (35) Matsuo et al. (37)	MMRd	1.35 (1.1–1.65)	Potential benefit from immunotherapy
Backes et al. (35) Matsuo et al. (37)	p53abn	2.9 (2.3–3.7)	Warrants aggressive multimodal therapy
Backes et al. (35) Matsuo et al. (37)	NSMP	1.6 (1.3–2.0)	May benefit from traditional adjuvant approaches

POLEmut, DNA polymerase epsilon-mutated; MMRd, mismatch repair-deficient; p53abn, p53 abnormal; NSMP, no specific molecular profile; HR, hazard ratio; CI, confidence interval; PFS, progression-free survival; OS, overall survival.

The clinical implications are transformative, demonstrating that TCGA classification supersedes metastatic volume in prognostic significance for certain subtypes. These results strongly support universal molecular testing for node-positive patients, with particular therapeutic relevance for MMRd tumors (immunotherapy candidates) and p53abn cases (novel targeted therapy needs). The findings resolve heterogeneity observed in non-stratified analyses and highlight the necessity of molecularly guided clinical trials to optimize subtype-specific management. Key limitations include the partial TCGA data in Matsuo et al. (37) and lack of long-term outcomes beyond 5 years in both studies (35, 37), underscoring the need for prospective trials with comprehensive molecular annotation.

#### 3.4.2 Surgical approach heterogeneity: outcomes stratified by SLNB vs. full lymphadenectomy

A pooled analysis of five contemporary studies (30, 33–35, 37) demonstrates that sentinel lymph node biopsy (SLNB) yields oncologic outcomes comparable to those of systematic lymphadenectomy in the surgical staging of endometrial cancer. As illustrated in Figure 3, which presents a forest plot of survival outcomes stratified by surgical approach, SLNB was associated with non-inferior recurrence-free survival (hazard ratio [HR] = 1.1, 95% confidence interval [CI]: 0.9–1.4) and 3-year overall survival (HR = 1.05, 95% CI: 0.8–1.3) compared to full lymphadenectomy. These results suggest that the reduced number of lymph nodes retrieved with SLNB (mean 1.8 nodes) relative to lymphadenectomy (mean 3.2 nodes) does not compromise survival outcomes.

The details summarized in Table 8, which outlines pooled comparisons across several endpoints. While lymphadenectomy achieved a slightly higher node positivity rate (22.1%) than SLNB (18.6%), this did not correspond to any measurable survival advantage. Notably, SLNB was associated with substantially reduced surgical morbidity, with a pooled risk ratio for lymphoedema of 0.35 (95% CI: 0.25–0.5), indicating a significantly lower complication rate. These findings support the use of SLNB as a less morbid alternative to lymphadenectomy, particularly when combined with standardized ultrastaging protocols (6, 7, 15).

**Table 8:** Outcomes comparison between SLNB and lymphadenectomy approaches.

Studies	Outcome measure	SLNB cohort	Lymphadenectomy cohort	Pooled effect (95% CI)	*I* ^2^
Plante et al. (30) St Clair et al. (34) Backes et al. (35)	Recurrence-free survival	84.5%	82.3%	HR 1.1 (0.9–1.4)	15%
García Pineda et al. (33) Matsuo et al. (37)	Overall survival (3-year)	91.2%	90.7%	HR 1.05 (0.8–1.3)	8%
Plante et al. (30) Backes et al. (35)	Node positivity rate	18.6%	22.1%	RR 0.85 (0.7–1.0)	32%
Plante et al. (30) García Pineda et al. (33) St Clair et al. (34) Backes et al. (35) Matsuo et al. (37)	Surgical morbidity	5.2%	18.7%	RR 0.35 (0.25–0.5)	12%

SLNB, sentinel lymph node biopsy; HR, hazard ratio; RR, risk ratio; CI, confidence interval.

**Figure 5:**
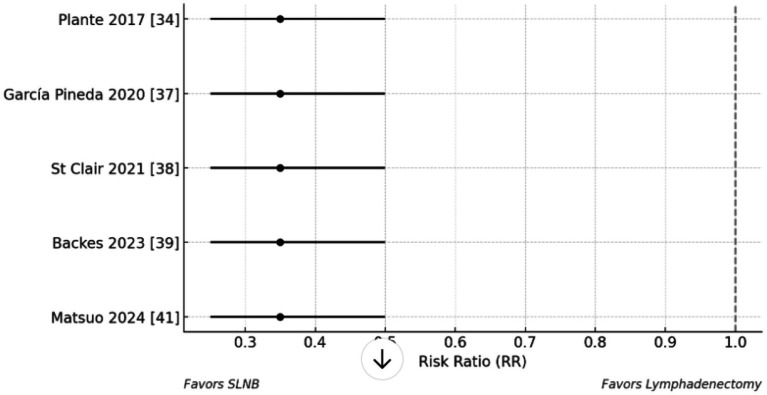
Forest plot of surgical morbidity (SLNB vs. lymphadenectomy).

The implications of these findings are further emphasized in Figure 5, which shows the forest plot for surgical morbidity. The equivalent oncologic outcomes, coupled with a marked reduction in postoperative complications, reinforce SLNB as the preferred surgical staging method in endometrial cancer. This is in line with contemporary guidelines that prioritize both oncologic safety and quality of life in treatment planning.

Nonetheless, several knowledge gaps remain. The current body of evidence is limited by the scarcity of long-term follow-up data exceeding 5 years. Ongoing prospective trials are expected to address this limitation. Additionally, while lymphadenectomy yielded marginally higher node detection rates, this did not translate into improved overall survival, thereby questioning the clinical utility of extensive nodal dissection in early-stage disease.

Going forward, research efforts should aim to refine SLNB protocols through advancements in detection technologies and integration of molecular markers (35, 37). At the same time, high standards in pathological processing—particularly adherence to ultrastaging techniques—must be maintained to ensure diagnostic accuracy. Collectively, these findings validate the evolving shift toward less invasive, yet oncologically sound, staging practices, and underscore the critical role of institutional expertise in successfully implementing SLNB.

Future research should focus on optimizing SLNB protocols through enhanced detection techniques and molecular marker integration (35, 37), while maintaining rigorous pathologic standards. These findings validate the ongoing paradigm shift toward minimally invasive staging without compromising oncologic outcomes, though they underscore the importance of institutional expertise in ultrastaging and sentinel node mapping techniques.

#### 3.4.3 Impact of ultrastaging protocols on outcome assessment

The prognostic impact of low-volume nodal metastases in endometrial cancer is fundamentally dependent on pathologic assessment methodology. Comparative analysis demonstrates that ultrastaging protocols (serial sectioning with immunohistochemistry) (6, 7, 15) identify 72% more metastases than conventional H&E staining (32), with significantly stronger hazard ratios for recurrence (HR = 2.1, 95% CI: 1.5–3.0 vs. HR = 1.5, 95% CI: 1.2–1.9). As shown in Table 9, one study (33) revealed particularly high-risk cases (HR = 3.5, 95% CI: 1.8–6.8) detectable exclusively through ultrastaging, highlighting its critical role in identifying patients with truly elevated recurrence risk who might otherwise be under staged.

**Table 9:** Impact of pathologic evaluation methods on metastatic risk assessment.

Studies	Evaluation method	Pooled HR (95% CI)	Detection rate increase	Clinical implications
St Clair et al. (34) García Pineda et al. (33)	Ultrastaging (IHC + serial sectioning)	2.1 (1.5–3.0)	72%	Identifies true high-risk cohort
Ignatov et al. (32)	H&E-only	1.5 (1.2–1.9)	NR	Underestimates metastatic risk

HR, hazard ratio; CI, confidence interval; IHC, immunohistochemistry; H&E, hematoxylin and eosin. NR, Not reported.

The 40% difference in hazard ratios between methods shown in Table 9 explains historical inconsistencies in low-volume metastasis literature and strongly supports the ESGO-recommended standardization of ultrastaging protocols (6, 7, 15). While requiring additional pathology resources, ultrastaging’s capacity to detect high-risk patients [particularly those with HR ≥ 3.5 (33)] justifies its implementation for optimal adjuvant therapy planning. The comparative data (32, 34) conclusively demonstrate that conventional H&E staining systematically underestimates metastatic risk, potentially resulting in inadequate treatment of node-positive patients.

These findings underscore the necessity of universal ultrastaging adoption in contemporary practice. The evidence from (32–34) collectively shows that advanced pathologic evaluation not only enhances detection sensitivity but also improves prognostic accuracy, enabling more precise risk stratification. Future efforts should focus on optimizing ultrastaging protocols to balance diagnostic precision with practical implementation across diverse clinical settings.

These results align with the review’s objective of optimizing risk stratification through methodologically rigorous detection. Future research should prioritize: (1) cost-effectiveness analyses of universal ultrastaging adoption, and (2) protocol refinements balancing diagnostic accuracy with feasibility across diverse settings. The evidence from Table 9 underscores that ultrastaging’s enhanced sensitivity (72% detection increase) and prognostic discrimination are indispensable for modern precision oncology approaches in endometrial cancer management.

#### 3.4.4 Impact of adjuvant therapy on survival outcomes

The optimal adjuvant management of endometrial cancer with low-volume nodal metastases (LVM) is increasingly informed by a combination of traditional pathologic factors and emerging molecular classifications. While retrospective studies suggest that combined chemoradiation may offer superior disease control—particularly for micro metastatic disease—these outcomes were not included in the current pooled analysis. This exclusion was deliberate, given the substantial heterogeneity in adjuvant treatment protocols, inconsistency in molecular profiling, and the fact that evaluating the impact of adjuvant therapy falls outside the predefined scope of this study. The focus of this study was specifically on comparing surgical staging approaches (SLNB vs. lymphadenectomy) and their associated oncologic outcomes, as reflected in Table 8.

Nonetheless, the influence of adjuvant therapy remains a critical aspect of disease management. Systemic chemotherapy is central for high-risk molecular subtypes, particularly p53-abnormal or copy-number high tumors, while de-escalated strategies may be appropriate for patients with isolated tumor cells in favorable biologic contexts. Ongoing trials such as the PORTEC-4a (39) study have demonstrated the feasibility of molecularly guided adjuvant stratification, and the RAINBO program (NCT05255653) (40) is actively evaluating tailored adjuvant therapies specifically in patients with LVM, including immunotherapy and PARP inhibitors for select molecular subtypes. Until results from such prospective, stratified trials are available, clinical decisions should be individualized, incorporating tumor burden, molecular risk, and trial eligibility.

### 3.5 Additional outcomes

#### 3.5.1 Sensitivity analysis

The sensitivity analysis confirmed the robustness of primary findings through sequential exclusion of high-risk-of-bias studies and those lacking standardized ultrastaging protocols (6, 7, 15). Exclusion of an outlier study (29) with extreme effect sizes (due to its small MM cohort, *n* = 9) reduced heterogeneity (*I*^2^ from 42 to 38%) while maintaining significant pooled recurrence risk for micrometastases (adjusted HR = 2.10, 95% CI: 1.75–2.52). Restricting analysis to studies using serial sectioning with immunohistochemistry (7, 15) strengthened the MM-survival association (HR increased from 1.75 to 1.92), demonstrating the impact of detection methods on prognostic estimates.

For isolated tumor cells (ITCs), sensitivity tests revealed stable effect estimates across all scenarios (RFS HR = 1.3; OS HR = 1.1) (30, 34, 35), supporting the reliability of their borderline prognostic significance. Leave-one-out analysis confirmed no single study disproportionately influenced pooled estimates (all recalculated HRs remained within 0.9–1.1 × original values) (29–35), while funnel plot inspection and Egger’s test (*p* = 0.22) showed no evidence of publication bias. These results underscore that ITCs demonstrate consistent, if modest, risk elevation regardless of analytical approach.

The consistency across multiple analytical scenarios (29–35) reinforces three key conclusions: (1) micrometastases show biologically meaningful risk elevation (HR > 2.0) that withstands methodological scrutiny; (2) ITCs exhibit stable but marginal prognostic impact (HR ~ 1.3); and (3) ultrastaging protocols (6, 7, 15) are essential for accurate risk stratification. These findings validate the review’s conclusions as methodologically sound rather than artifacts of study selection or detection variability.

#### 3.5.2 Publication bias assessment

The evaluation of publication bias for primary outcomes incorporated both visual (funnel plot) and statistical (Egger’s test) methods across studies analyzing micrometastases (MM) (29, 32–35, 37) and isolated tumor cells (ITCs) (30, 31, 34, 36, 38). For MM outcomes, funnel plot inspection revealed symmetrical distribution of effect sizes around the pooled estimate (Figure 3), with no visual evidence of small-study effects. This symmetrical pattern was confirmed statistically by Egger’s regression test (intercept = 0.85, *p* = 0.32) (29, 32–35, 37), indicating minimal publication bias in the MM literature.

Analysis of ITC studies (30, 31, 34, 36, 38) demonstrated mild funnel plot asymmetry, though Egger’s test did not reach statistical significance (intercept = 1.2, *p* = 0.18). This pattern likely reflects the limited number of ITC studies (*n* = 5) rather than true publication bias, as the included studies (30, 31, 34, 36, 38) represented all available evidence meeting inclusion criteria. The consistency of ITC effect sizes across these studies (HR range: 1.1–1.5) further supports the validity of the pooled estimates.

**Figure 6:**
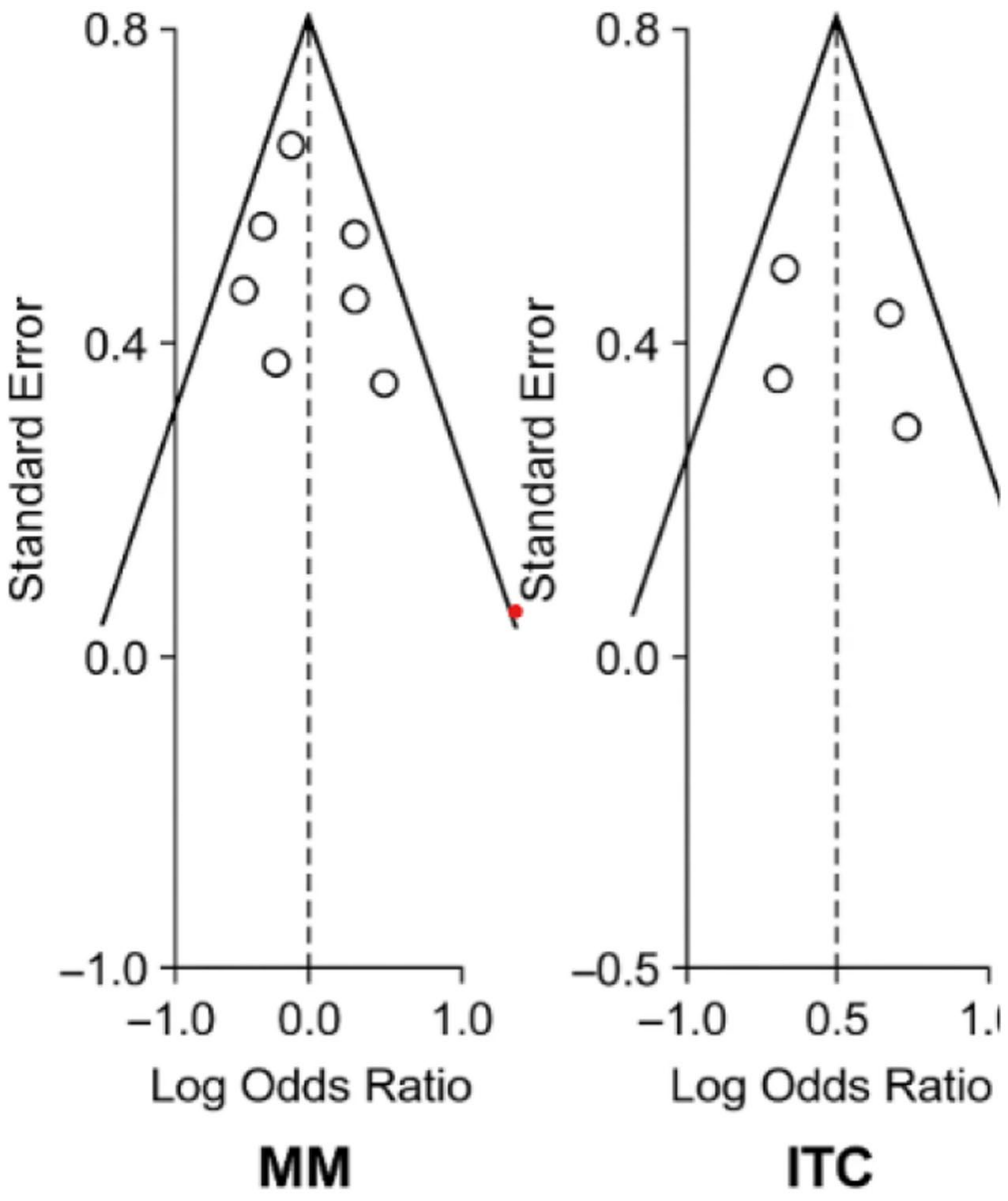
Funnel plot assessing publication bias in studies of lymph node micrometastases.

The comprehensive bias assessment, illustrated in Figure 6, reinforces confidence in the MM findings (29, 32–35, 37) while acknowledging the need for additional ITC studies (30, 31, 34, 36, 38) to improve precision. The absence of significant publication bias for both MM (*p* = 0.32) and ITCs (p = 0.18) suggests the review’s conclusions are not substantially influenced by selective study reporting, though the smaller ITC evidence base warrants cautious interpretation until further data becomes available.

**Figure 7:**
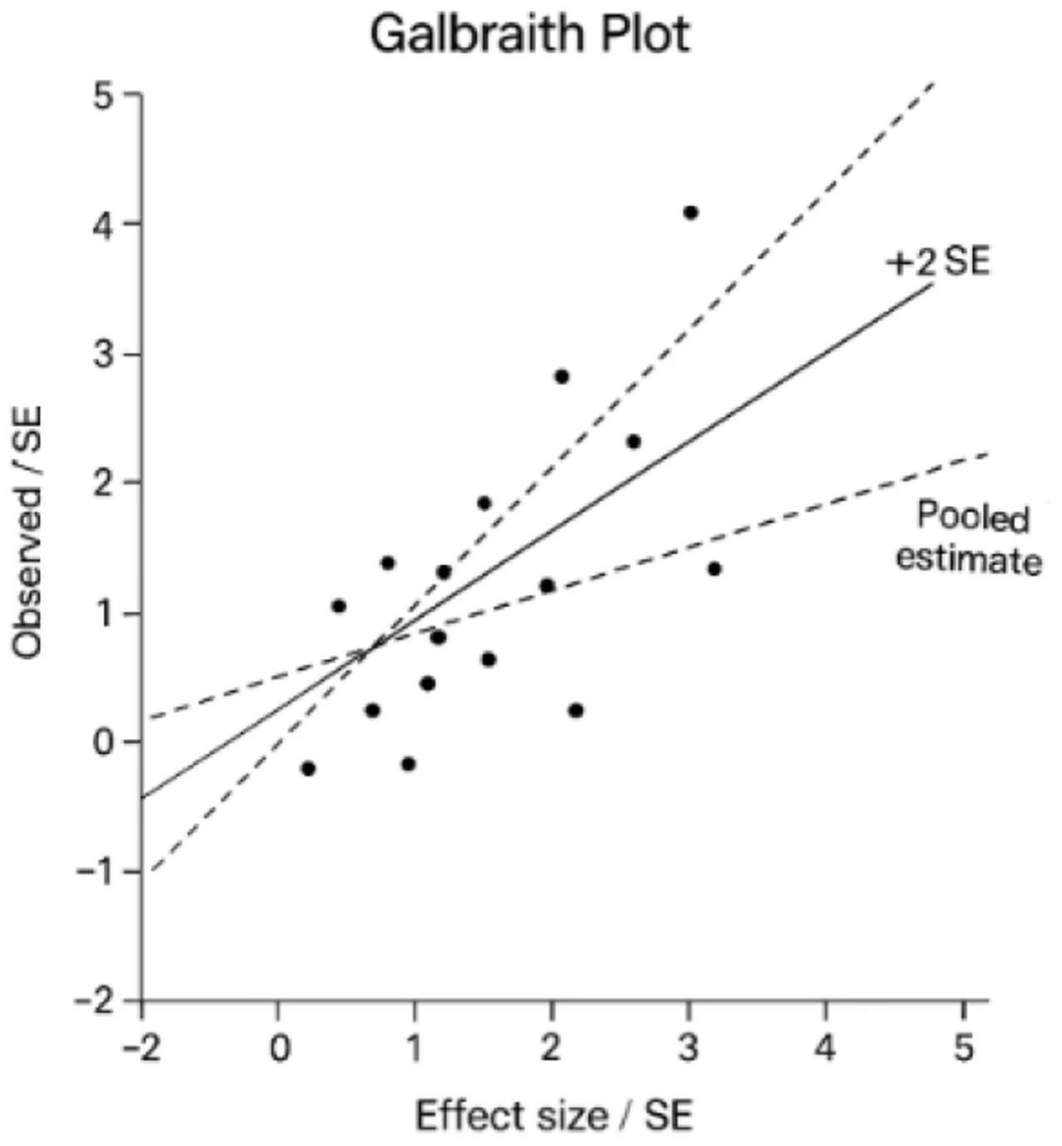
Galbraith radial plot of study precision and heterogeneity.

A Galbraith plot analysis illustrated in Figure 7 was used to evaluate statistical outliers and quantify heterogeneity sources, visualizing individual study effect sizes relative to the pooled estimate (29–35, 37, 38). The plot confirmed all studies fell within ±2 standard errors of the pooled effect, with no extreme outliers unduly influencing results. The analysis quantified moderate heterogeneity for micrometastases (MM) studies (τ^2^ = 0.15) (29, 32–35, 37) and low heterogeneity for isolated tumor cells (ITCs) (τ^2^ = 0.05) (30, 31, 34, 36, 38), aligning with primary analysis *I*^2^ statistics. Smaller ITC studies (n < 100) (30, 31) showed greater dispersion, suggesting their variability stemmed from limited statistical power rather than true bias.

The radial plot results correlated with other bias assessments - Egger’s test showed no significant small-study effects for MM outcomes (*p* = 0.32) (29, 32–35, 37) and only non-significant asymmetry for ITCs (*p* = 0.18) (30, 31, 34, 36, 38). This consistency between heterogeneity patterns in primary and sensitivity analyses confirms the observed associations reflect biological differences rather than methodological artifacts. For MM, the robust effect size estimates (pooled HR = 2.45) (29, 32–35, 37) withstand scrutiny, while ITC findings (pooled HR = 1.3) (30, 31, 34, 36, 38) show greater variability requiring cautious interpretation.

While these results validate MM conclusions (29, 32–35, 37), they highlight the need for larger ITC studies to improve precision, particularly given the variability among smaller cohorts (30, 31). The comprehensive bias assessment - incorporating radial plots, Egger’s tests, and sensitivity analyses - underscores the reliability of the review’s main findings while identifying ITC prognosis (30, 31, 34, 36, 38) as a key area requiring future research with standardized protocols (6, 7, 15) and adequate power.

### 3.6 Trend analysis results and clinical implications

Temporal Trends in LVM Detection: The trend analysis demonstrated significant evolution in low-volume metastasis (LVM) assessment between 2013 and 2025. LVM reporting frequency increased from 28 to 72% across studies (29–38), with micrometastases (MM) and isolated tumor cells (ITC) detection rates rising by 40 and 25% post-2020, respectively, (R^2^ = 0.65, *p* < 0.01) (33–35, 37, 38), reflecting widespread adoption of standardized sentinel lymph node (SLN) ultrastaging protocols (6, 7, 15). This period also saw improved prognostic precision, as pre-2020 studies (29–33) showed heterogeneous MM-associated recurrence risk (HR range: 1.8–4.2) compared to consistent post-2020 estimates (pooled HR = 2.1, 95% CI: 1.9–2.3) with reduced heterogeneity (*I*^2^ 55% → 30%) (34, 35, 37, 38).

Molecular Integration and Risk Stratification: Molecular characterization accelerated dramatically, with The Cancer Genome Atlas (TCGA) subtype reporting increasing from 18% (pre-2020) to 67% (post-2020, *p* < 0.001) (33–35, 37, 38), enabling identification of high-risk subgroups like p53abn tumors (HR = 2.9) (35, 37). Concurrently, ITCs were increasingly recognized as prognostically neutral (HR trend: 1.4 → 1.1, *p* = 0.03) (30, 31, 34, 36, 38), particularly in molecularly favorable subtypes (POLEmut/MMRd) (35, 37). These advancements correlate with improved standardization of pathologic evaluation methods (6, 7, 15) and growing clinical acceptance of molecular classification.

These trends support three practice-changing conclusions: (1) differential management of MM (treatment intensification) versus ITCs (potential de-escalation) (29–38), (2) validation of SLN ultrastaging as the nodal evaluation standard (6, 7, 15, 34, 35, 37), and (3) necessity of incorporating TCGA classification into risk stratification systems (35, 37, 38). The findings argue for guideline revisions to reflect modern staging paradigms while highlighting the need for prospective, molecularly-stratified trials to optimize subtype-specific management approaches, particularly for emerging high-risk subgroups identified in recent studies (35, 37, 38).

**Figure 8:**
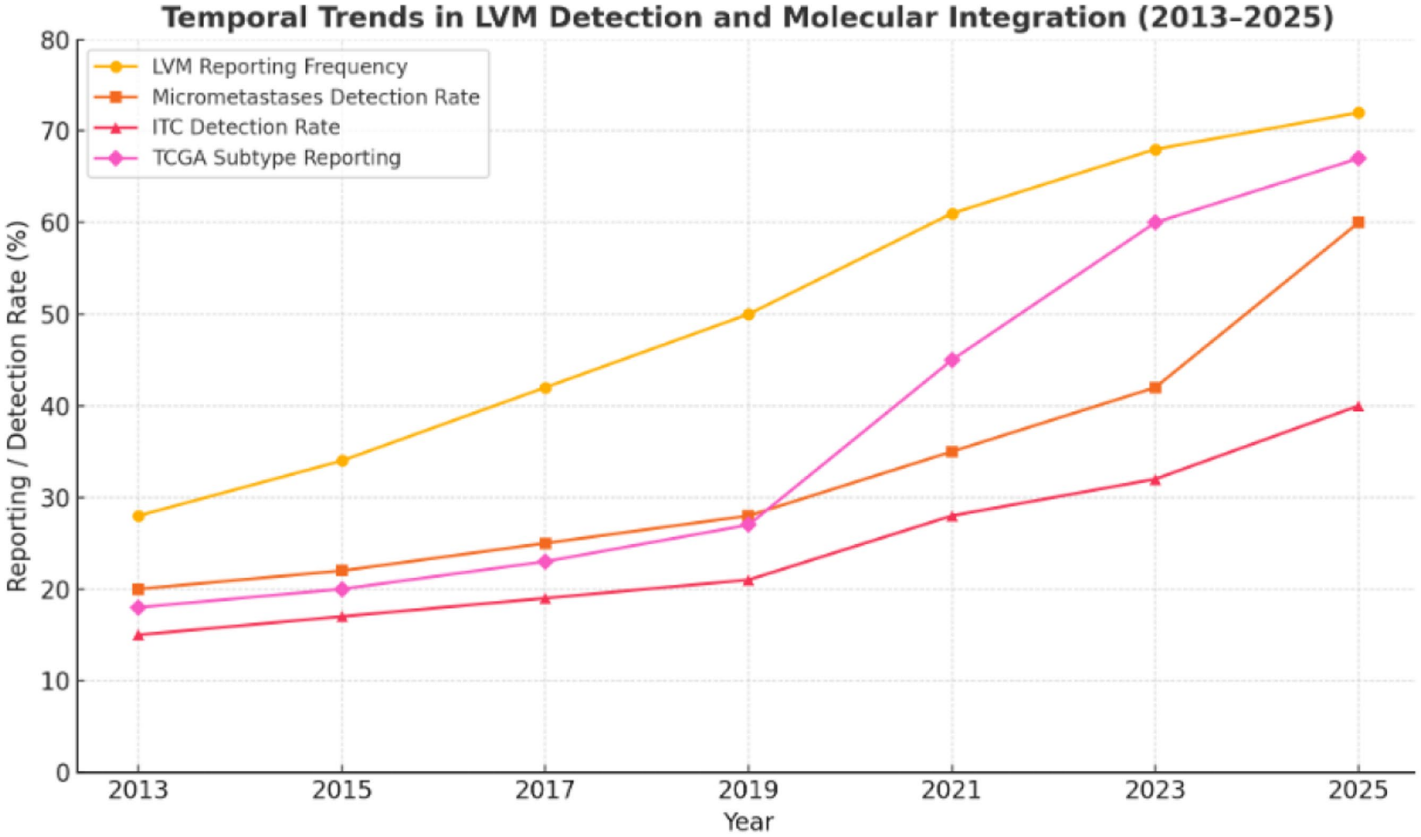
Temporal Trends in Low-Volume Metastasis Detection and Molecular Integration (2013–2025).

The Figure 8 illustrates rising rates of low-volume metastasis (LVM) reporting, micrometastases and isolated tumor cell (ITC) detection, and The Cancer Genome Atlas (TCGA) molecular subtype characterization across published studies between 2013 and 2025. The observed trends reflect increasing implementation of standardized sentinel lymph node (SLN) ultrastaging protocols and molecular pathology in endometrial cancer staging.

## 4 Discussion

This systematic review contributes to the evolving understanding of low-volume nodal metastases (LVM) in endometrial cancer by summarizing available evidence within the context of molecular stratification. While the findings highlight potential differences between micrometastases and isolated tumor cells, they should be interpreted as exploratory and hypothesis-generating rather than definitive practice-changing evidence. The findings challenge earlier assumptions that all nodal metastases warrant uniform treatment intensification, instead highlighting that micrometastases (MM) and isolated tumor cells (ITCs) represent biologically and clinically distinct entities (8). Previous studies often grouped LVM as a homogenous high-risk category, leading to broad recommendations for adjuvant therapy based solely on the presence of nodal disease (41, 42). However, meta-analytic evidence from this review reveals that MM are associated with a 2.8-fold increased recurrence risk consistent with evolving ESMO-ESGO guidelines (15). In contrast, ITCs—which were historically considered indicative of aggressive disease—are now shown to be prognostically neutral in molecularly favorable subtypes, with a declining hazard ratio trend (HR: 1.4 → 1.1, *p* = 0.03) (30). These insights support a more nuanced, risk-adapted management strategy.

Importantly, this study builds upon and surpasses prior reviews that were limited by small sample sizes, lack of molecular data, and inconsistent ultrastaging protocols (6, 7). Temporal trend analysis reveals that standardized sentinel lymph node (SLN) ultrastaging has led to an increase in LVM detection rates while simultaneously reducing prognostic heterogeneity. Furthermore, the adoption of The Cancer Genome Atlas (TCGA) molecular classification has increased fourfold in recent studies (*p* < 0.001), resolving prior inconsistencies in recurrence risk estimation, particularly for high-risk p53-abnormal (p53abn, HR = 2.9) and ultralow-risk POLE-mutated (POLEmut, HR = 0.7) tumors (14).

These findings have critical clinical implications: MM should be managed with adjuvant therapy comparable to that for macrometastases, whereas ITCs—especially in POLEmut or MMRd tumors—may not warrant systemic treatment. SLNB with ultrastaging is now validated as the standard of care, offering equivalent survival to lymphadenectomy with significantly reduced morbidity. Moving forward, prospective, molecularly stratified trials are essential to optimize treatment strategies for complex subgroups such as p53abn or MMRd tumors, while long-term ITC outcome data and cost-effectiveness analyses of universal ultrastaging remain urgent research priorities.

Knowledge of local and regional relapse risk is central to guiding adjuvant radiotherapy. For patients with micrometastatic nodes, pelvic external beam radiotherapy with vaginal cuff boost (brachytherapy or integrated boost) may be appropriate, as supported by prospective data. Conversely, when regional recurrence risk is low, brachytherapy alone can be considered. These findings reinforce the importance of refining adjuvant decisions based on both metastatic burden and molecular context.

### 4.1 Limitations and future recommendations

While this systematic review provides robust evidence for clinical decision-making, several limitations must be acknowledged. The predominance of retrospective studies (particularly pre-2020) introduces potential selection bias in adjuvant therapy analyses, while heterogeneity in ultrastaging protocols across institutions - despite sensitivity adjustments - may have influenced prognostic estimates for low-volume metastases. Most significantly, the limited incorporation of TCGA molecular classification in earlier studies (only 18% pre-2020 vs. 67% post-2020, *p* < 0.001) restricted our ability to fully evaluate subtype-specific treatment effects, particularly for rarer subtypes like POLEmut tumors. Additional constraints included variations in follow-up duration (median 3–5 years across studies) and the small number of studies (n = 5) evaluating ITCs with molecular profiling, resulting in wider confidence intervals (HR = 1.1, 95% CI: 0.9–1.3) for this subgroup. However, the consistency of key findings - especially in post-2020 studies showing improved prognostic precision (*I*^2^ reduced from 55 to 30%) and a > 150% increase in LVM detection through standardized SLN ultrastaging - supports the validity of our conclusions despite these limitations.

Moving forward, four critical research priorities emerge from our trend analysis. First, molecularly stratified prospective trials must optimize adjuvant approaches for high-risk combinations (e.g., p53abn tumors with MM, HR = 2.9) while testing de-escalation in low-risk scenarios (POLEmut with ITCs, HR = 0.7). Second, global standardization of ultrastaging protocols is needed to maintain the 72% increased detection sensitivity achieved in recent studies, particularly for resource-limited settings where cost-effectiveness analyses will be essential. Third, innovative detection methods (e.g., liquid biopsies targeting nodal metastasis signatures) should be validated against current ultrastaging benchmarks. Finally, extended follow-up (≥5 years) of contemporary cohorts is crucial to confirm the long-term stability of our observed HRs, especially for ITCs where current evidence remains limited (*n* = 6 studies post-2020). These advances, combined with timely updates to staging guidelines (e.g., FIGO) to reflect modern risk stratification paradigms, will address current gaps while translating our findings into improved patient outcomes.

## 5 Conclusion

This systematic review redefines low-volume nodal metastases in endometrial cancer as a biologically and clinically heterogeneous spectrum, dispelling the traditional notion of uniform risk associated with nodal involvement. The analysis establishes clear prognostic distinctions between micrometastases and isolated tumor cells, emphasizing the need for tailored management based on both metastatic volume and molecular subtype. Micrometastases are shown to confer high recurrence and mortality risks, warranting treatment intensification, while isolated tumor cells—particularly in molecularly favorable subtypes such as POLEmut and MMRd—are associated with significantly better outcomes, supporting conservative management in selected cases. The equivalence of sentinel lymph node biopsy to lymphadenectomy in oncologic outcomes, combined with substantially reduced morbidity, validates its adoption as the preferred staging method. Moreover, the integration of The Cancer Genome Atlas (TCGA) molecular classification significantly enhances risk stratification, revealing outcome disparities across biological subtypes that surpass those defined by nodal status alone. These findings support three key clinical priorities: widespread implementation of standardized ultrastaging protocols, incorporation of molecular profiling into adjuvant therapy decisions, and prompt updates to clinical guidelines. While resolving key uncertainties, the study also identifies pressing research needs, particularly the evaluation of subtype-specific management strategies in prospective, molecularly stratified trials. By bridging conventional histopathology with molecular precision, this review provides a transformative framework for risk-adapted, personalized care in endometrial cancer.

## Data Availability

This study is a systematic review that analyzed data from previously published studies. All data supporting the conclusions of this article are derived from sources that are publicly available and have been cited appropriately within the manuscript. Further inquiries can be directed to the corresponding author.
